# Short note on reflective writing

**DOI:** 10.6026/973206300210159

**Published:** 2025-02-28

**Authors:** Himel Mondal, Shaikat Mondal

**Affiliations:** 1 Department of Physiology, All India Institute of Medical Sciences, Deoghar, Jharkhand, India; 2 Department of Physiology, Raiganj Government Medical College and Hospital, West Bengal, India

**Keywords:** Reflective writing, medical education, critical thinking, self-assessment, professional development

## Abstract

Individual reflective writing significantly enhances learning outcomes. Reflective writing fosters critical thinking, self-assessment and clinical reasoning skills that are essential for the professional development of medical students. However, implement reflective writing among Indian medical college student face challenges due to traditional curricula with inadequate faculty training. Hence, institutions must integrate reflective writing practices into their curricula and subsequently train educators.

## Background:

Individual reflective writing has potential to enhance Indian medical students' learning outcomes by fostering self-assessment, critical thinking, and metacognition, which are crucial for clinical reasoning and decision-making [1, 2]. Reflective writing can serve as a powerful tool to enhance professional development as medical education evolves towards a more student-centered and experiential approach [3]. However, while the benefits are clear, there are notable practical challenges in implementing reflective writing on a large scale in Indian medical institutions. The traditional medical curriculum in India is heavily focused on didactic lectures and routine learning. A sudden change is practically not feasible due to limited manpower, poor infrastructure and lack of training [4]. Hence, there is little room for innovative learning methods like reflective writing in a tight schedule of completing the course in limited time. With packed schedules that include theoretical classes, clinical postings and extracurricular activities, both students and faculty often find it difficult to dedicate the time required for reflective writing exercises. Medical students are under constant pressure to perform in exams and this often leads to a prioritization of content retention over critical reflection. Moreover, many faculty members may not be adequately trained to facilitate reflective writing exercises or guide students in the art of self-assessment [5]. This lack of training creates a gap between the potential benefits of reflective writing and its practical execution in classrooms. Mitigating the challenges of implementing reflective writing, especially in Indian medical colleges, requires a multi-faceted approach. Institutions must integrate reflective writing into the existing curriculum by allocating dedicated time for reflection during clinical rotations and feedback sessions. Faculty development programs are crucial to train educators in guiding students through the reflective process and assessing their reflections effectively [6]. Additionally, leveraging digital tools such as online journals or e-portfolios can streamline the reflective writing process [7]. Encouraging a culture of open communication and self-reflection within the academic environment can help reduce the resistance students may feel. They should realize that it is a valuable tool for personal and professional growth rather than an added burden. In this context, we would like to share top ten views of reflective writing for medical teachers and it is shown in Table 1 [3, 8]. Jiwane et al. highlighted the significance of reflective writing in medical education [1]. It shows strong evidence of its effectiveness in improving learning outcomes. Hence, Indian medical colleges should embrace reflective writing as a fundamental part of their educational framework.

## Figures and Tables

**Table 1:** Top ten tenets of reflective writing

**Tenet**	**Description**
Self-awareness	Introspection to understand personal strengths, weaknesses and behaviors.
Critical thinking	Encourages analyzing experiences, questioning assumptions and thinking deeply.
Continuous learning	Viewing every experience as an opportunity for growth and improvement.
Emotional intelligence	Focuses on recognizing and understanding emotional responses.
Metacognition	Promotes thinking about one's own thought processes and learning strategies.
Action-oriented reflection	Reflection leads to practical outcomes, encouraging individuals to apply insights and lessons learned to future behavior.
Integration of theory and practice	Bridges the gap between academic knowledge and real-world application.
Structured reflection	Ensuring thorough and systematic analysis of experiences.
Honesty and openness	Encourages genuine reflection by acknowledging mistakes and challenges.
Development of professional identity	Helps individuals shape their professional identity by reflecting on their values, roles and aspirations.
